# Supplementary material for: Effect of Docosahexaenoic Acid (DHA) at the Enteric Level in a Synucleinopathy Mouse Model
Source: Nutrients. 2021 Nov 24;13(12):4218. doi: 10.3390/nu13124218 (PMC8703327; doi:10.3390/nu13124218)
Supplement: Supplementary file 1 [file nutrients-13-04218-s001.zip › FiguresSuppl.pdf]

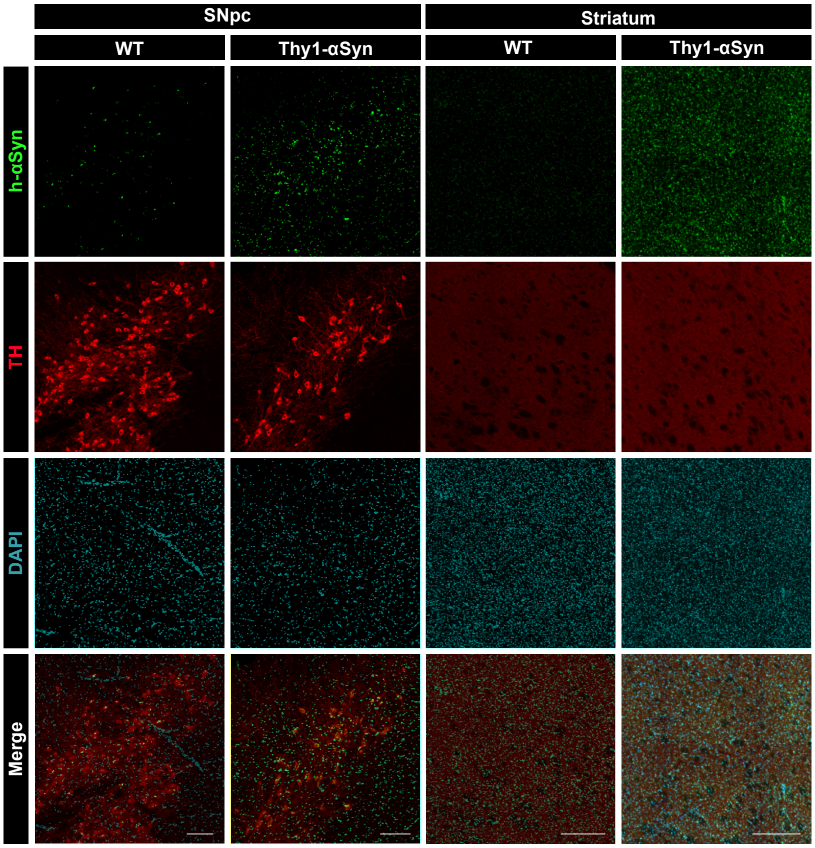

Figure S1. Detection of αSyn in murine Thy1-αSyn brains. Immunofluorescence for wild-type human αSyn and TH in brain slices from both wildtype littermates and Thy1-αSyn mice. Sections selected at the striatum and SNpc level revealed the presence of αSyn only in the Thy1-αSyn model. Scale bar = 30 µm. **Abbreviations:** h-αSyn, human alpha-synuclein; SNpc, substantia nigra *pars compacta*; TH, tyrosine hydroxylase.

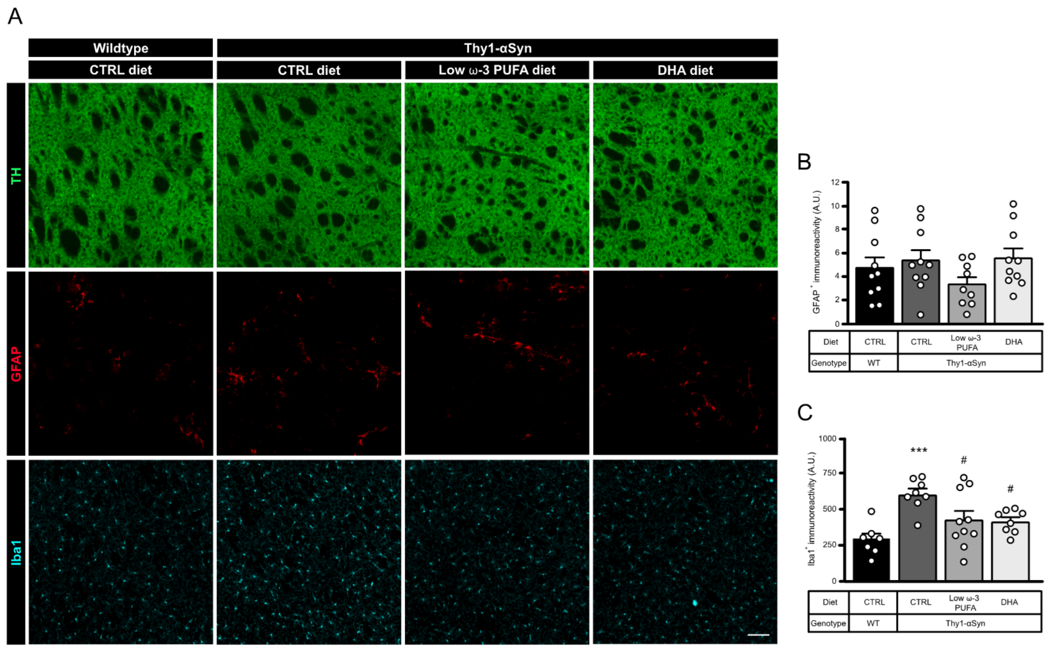

Figure S2. A DHA-rich diet prevents inflammation in Thy1-αSyn mice brain. A. Photomicrographs of TH (green), GFAP+ astrocytes (red) and Iba1+ microglia (cyan) immunofluorescence in the striatum of wild-type and Thy1-αSyn mice. Scale bar = 100 µm. **B.** Quantification of GFAP+ astrocytes immunoreactivity shows no significant difference between each group (one-way ANOVA: $p = 0.245$). **C**. Quantification of Iba1+ macrophages by immunoreactivity reveals an increase of these cells in Thy1-αSyn mice compared to wild-type littermates, both on control diet. Interestingly, transgenic mice on both low ω-3 PUFA or DHA-enriched diet show a decrease of macrophages compared to those on control diet (one-way ANOVA: $p = 0.0011$). Tukey's post-hoc tests: $^{***}p < 0.001$ compared with wild-type mice; $^{\#}p < 0.05$ compared with Thy1-αSyn on control diet. **Abbreviations:** CTRL, control; DHA, docosahexaenoic acid; GFAP, glial fibrillary acidic protein; Iba1, ionized calcium-binding adapter molecule 1; TH, tyrosine hydroxylase.
