# Supplementary material for: Effect of Docosahexaenoic Acid (DHA) at the Enteric Level in a Synucleinopathy Mouse Model
Source: Nutrients. 2021 Nov 24;13(12):4218. doi: 10.3390/nu13124218 (PMC8703327; doi:10.3390/nu13124218)
Supplement: Supplementary file 1 [file nutrients-13-04218-s001.zip › TableS1-DietaryTreatments.pdf]

Table S1. Description of dietary treatments.

	Control	Low DHA	High DHA
	Medium LNA<br>No DHA	Low LNA<br>No DHA	Low LNA<br>High DHA
**Protein (% w/w)**	20.3	20.3	20.3
**Carbohydrate (% w/w)**	66.0	66.0	66.0
**Fat (% w/w)**	5.0	5.0	5.0
**Calorie per diet weight kcal/g**	3.9	3.9	3.9
**Ingredients (g/kg)**			
Casein	200	200	200
DL-Methionine	3	3	3
Corn Starch	150	150	150
Sucrose	500	500	500
Cellulose, BW200	50	50	50
Corn Oil	30	25	10
Safflower Oil	0	25	19
DHA powder KSF58	0	0	50
Soybean Oil	10	0	0
Canola oil	10	0	0
**Fatty acids (g/kg)**[1]			
Total n-3 PUFA	1.43	0.31	7.80
LNA	1.42	0.28	0.14
EPA	0	0	0.26
DHA	0	0	7.20
Total n-6 PUFA	21.20	30.29	18.42
Linoleic acid	21.10	30.14	16.65
Arachidonic acid	0	0	0.06
n-6:n-3 PUFA ratio	14.82	97.70	2.36

Abbreviations and notes: n-3 and n-6 = n-3 and n-6 polyunsaturated fatty acids (PUFA) DHA: docosahexaenoic acid (22:6n-3) from DHA microencapsulated powder (Martek/DSM) EPA: eicosapentaenoic acid (20:5n-3) This formulation contains 1:25 ratio of to DHA LNA: alpha-linolenic acid (18:2 n-3) (from soybean oil) [1] Determined within the diet by gas chromatography.
