# Supplementary material for: Effect of Docosahexaenoic Acid (DHA) at the Enteric Level in a Synucleinopathy Mouse Model
Source: Nutrients. 2021 Nov 24;13(12):4218. doi: 10.3390/nu13124218 (PMC8703327; doi:10.3390/nu13124218)
Supplement: Supplementary file 1 [file nutrients-13-04218-s001.zip › TableS2Antibodies.pdf]

Table S2. Primary and secondary antibodies.

**Primary antibodies**	**Host**	**Company**	**Catalog #**	**Dilution**
Human α-Synuclein	Rabbit	Abcam	Ab138501	1/250
ChAT	Goat	Millipore	Ab144P	1/100
GFAP	Mouse	Millipore	MAB360	1/500
GLP-1	Rabbit	Invitrogen	PA5-79303	1/750
GPR120	Rabbit	LifeSpan Biosciences	LS-B11842	1/100
HuC/HuD	Mouse	Invitrogen	A-21271	1/200
Iba1	Rabbit	Wako	019-19741	1/250
MHCII	Rat	BD Pharmingen	556999	1/500
Nrf2	Rabbit	Abcam	Ab62352	1/100
TH	Sheep	Abcam	Ab113	1/200
**Secondary antibodies**	**Host**	**Company**	**Catalog #**	**Dilution**
Anti-Goat Alexafluor 488	Donkey	Life Technologies	A11055	1/1000
Anti-Mouse Cy3	Goat	Life Technologies	A10521	1/1000
Anti-Rabbit Alexafluor 488	Donkey	Life Technologies	A21206	1/1000
Anti-Rabbit Alexafluor 555	Donkey	Life Technologies	A31572	1/1000
Anti-Rabbit Alexafluor 647	Donkey	Life Technologies	A31573	1/1000
Anti-Rat Alexafluor 488	Donkey	Life Technologies	A21208	1/1000
Anti-Sheep Alexafluor 488	Donkey	Life Technologies	A11015	1/1000
Anti-Sheep Cy3	Donkey	Millipore	AP184C	1/800
Anti-Rabbit Alexafluor 647	Donkey	Life Technologies	A31573	1/1000
