# Supplementary material for: Effect of Docosahexaenoic Acid (DHA) at the Enteric Level in a Synucleinopathy Mouse Model
Source: Nutrients. 2021 Nov 24;13(12):4218. doi: 10.3390/nu13124218 (PMC8703327; doi:10.3390/nu13124218)
Supplement: Supplementary file 1 [file nutrients-13-04218-s001.zip › TableS3MassTransitionECB.pdf]

Table S3. Specific mass transitions of endocannabinoids and related mediators**.**

Lipid	Internal Standard Used	Q1 → Q3	Retention Time (min)
LEA-d4	-	328.20 → 66.10	11.48
LEA	LEA-d4-	324.30 → 62.05	11.53
DHEA-d4		376.30 → 66.0	11.62
DHEA	DHEA-d4	372.20 → 62.20	11.67
DPEA(n-3)	DHEA-d4	374.0 → 62.2	12.50
DPEA(n-6)	DHEA-d4	374.2 → 62.1	12.30
AEA-d4		352.10 → 66.20	11.64
AEA	AEA-d4	348.30 → 333.20	11.69
PEA-d4		304.00 → 62.30	12.01
PEA	PEA-d4	300.30 → 62.10	12.04
OEA-d4		330.20 → 66.10	12.61
OEA	OEA-d4	326.00 → 62.05	12.63
SEA-d3		331.10 →62.30	13.35
SEA	SEA-d3	328.00 → 62.10	13.64
1-AG-d5		384.50 → 287.20	12.64
1/2-AG	1-AG-d5	379.30 → 287.25	12.54
1/2-OG	1-AG-d5	357.20 → 264.10	13.27
1/2-SDG	1-AG-d5	351.2 → 259.2	10.10
1-LG-d5		360.00 → 263.30	12.63
1/2-LG	1-LG-d5	355.20 → 263.30	12.50
1-EPG-d5		382.20 → 285.25	11.67
1/2-EPG	1-EPG-d5	377.10 → 285.25	11.66
1-DHG-d5		408.2 → 311.20	12.52
1/2-DHG	1-DHG-d5	403.20 → 311.20	12.50
1-DPG-d5		410.3 → 313.3	12.80
1/2-DPG(n-3)	1-DPG-d5	405.2 → 313.3	12.60

Abbreviations: AEA, anandamide; AG, arachidonoyl glycerol; DHEA, docosahexaenoyl ethanolamide; DHG, docosahexaenoyl glycerol; DPEA, docosapentaenoyl ethanolamide; DPG, dipalmitoyl glycerol; EPG, eicosapentaenoyl glycerol; LEA, linoleoyl ethanolamide; LG, linoleoyl glycerol; OEA, oleoyl ethanolamide; OG, oleoyl glycerol; PEA,

palmitoyl ethanolamide; SEA, stearoyl ethanolamine; SDG, 1-stearoyl-2-docosahexaenoyl glycerol.
